# The effect of Peyton’s four-step method for teaching point-of-care ultrasound psychomotor skills: an experimental study

**DOI:** 10.1186/s13089-025-00466-w

**Published:** 2025-11-11

**Authors:** Michael Breunig, Ryan Kingsley, Darrell Schroeder, Jason Kraus, Corbin Plooster, Tiffany Galush, Laura Boldenow, Taryn Ragaisis, Hannah Regan, Will M. Schouten, Raheel Shafay, Meltiady Issa, Deanne T. Kashiwagi

**Affiliations:** Mayo Clinic, 200 1st Street SW, 55905 Rochester, MN USA

**Keywords:** Point-of-care ultrasound, POCUS, Bedside ultrasound, Psychomotor skills, Medical education, Physician assistant, Competency

## Abstract

**Background:**

Medical education commonly utilizes the “see one, do one” two-step approach for teaching psychomotor skills; however, recent evidence suggests that Peyton’s four-step method leads to superior learning. There is limited evidence, and almost no high-quality studies, specifically evaluating the effect of Peyton’s Four‑Step method on long-term retention of ultrasound/POCUS procedural skills. The purpose of this research project was to evaluate the effectiveness of Peyton’s four-step method on teaching the POCUS psychomotor skills of image acquisition to novice learners. Additionally, this research project assessed the influence of Peyton’s four-step method at three different points in time during the skill acquisition phase, in the setting of ongoing deliberate skill practice.

**Methods:**

A single-blinded, repeated measures interventional study based on experimental design was completed. Physician Assistant students from one large academic medical center were randomized into a control group (using the two-step method) and intervention group (using Peyton’s four-step method). Students were taught POCUS of the aorta, bladder, heart, lungs, and kidneys. Students’ POCUS skills were assessed during the immediate, intermediate, and delayed learning phases. At each assessment, an organ-specific score and a total score were obtained. Scores were compared using a Wilcoxon rank sum test. An ordinal logistic regression analysis was performed using a generalized linear mixed model with a multinomial distribution and cumulative logit link function to assess the overall effect of Peyton’s four-step method.

**Results:**

Students who were taught using Peyton’s method were found to have an increased likelihood of higher total scores compared to those taught using usual instruction (OR = 4.2, *p* = 0.003). Peyton’s method was found to have increased likelihood of higher scores for cardiac (OR = 2.3, *p* = 0.032), lung (OR = 2.5, *p* = 0.034), and kidney (OR = 3.0, *p* = 0.015). Student performance statistically improved with Peyton’s four-step method during the immediate (*p* = 0.031) and delayed (*p* = 0.011) skill acquisition phases, but not in the intermediate phase.

**Conclusion:**

Peyton’s four-step method improves overall psychomotor skill acquisition for POCUS. Peyton’s four-step method specifically improved psychomotor skills in the immediate skill acquisition phase and the delayed skill acquisition phase. The benefit of Peyton’s four-step method was more prominent in POCUS applications with higher complexity.

## Background

Point-of-Care Ultrasound (POCUS) is an ultrasound examination, performed by a clinician, at a patient’s bedside to answer a specific diagnostic or therapeutic question [1, 2]. In contrast to formal consultative ultrasonography, POCUS is quicker and expedites care [3, 4]. POCUS has many clinical applications. It can be utilized to evaluate specific organ systems, such as the aorta, bladder, heart, lungs, kidneys, soft tissues, and upper and lower extremity deep veins [2, 5–9]. Additionally, multi-organ system protocols have been developed to evaluate patients with respiratory failure, trauma, and shock [6, 7, 10]. Given its demonstrated utility, POCUS is increasingly being integrated into medical training, including medical school, residency, and Physician Assistant (PA) programs.^1^ Thus, understanding the most effective ways to teach POCUS has widespread importance in medical education.

POCUS involves concurrent utilization of both the cognitive and psychomotor domains of learning. POCUS training and assessment can generally be split into the components of basic knowledge (anatomy, physiology, ultrasound utilization), image acquisition (the psychomotor domain of utilizing the ultrasound to obtain high quality images), image interpretation (identifying normal findings and pathology on ultrasound images), and clinical integration (utilizing the generated images and interpretation to advance patient care) [2, 11]. Psychomotor image acquisition is an important distinguishing factor for POCUS from consultative ultrasonography, and image acquisition is required prior to image interpretation in clinical practice. Additionally, poor image acquisition can impair image interpretation or lead to incorrect interpretation, adversely impacting care [2, 11]. Thus, psychomotor image acquisition is of paramount importance in POCUS education.

Psychomotor skill development requires repeated, deliberate practice to achieve mastery, which is true for POCUS image acquisition as well [12–17]. A recent systematic review demonstrated that while different POCUS applications require different amounts of practice, all require some degree of deliberate practice to improve performance [13]. Notably, more complicated applications, such as cardiac POCUS, and to a lesser extent aorta, lung, and renal POCUS, require more deliberate practice to achieve mastery than simpler applications, such as bladder POCUS [13, 14].

Understanding the most effective way to teach psychomotor skills has significant implications in medical education. Improved psychomotor skill acquisition can lead to increased educational efficiency and potentially better patient outcomes. Psychomotor skills training in medical education programs have historically adopted a “see one, do one” two-step approach first proposed by Dr. Halsted in 1904, utilizing the steps of:

1. Demonstration—The entire skill is performed in real time, with limited commentary or teaching.
2. Performance—The learner completes the skill while receiving feedback from the teacher [18].

Despite its development over one hundred years ago, Halsted’s two-step approach is still commonly utilized in medical education [19].

Dr. JWR Peyton proposed a method for teaching clinical skills in 1998 utilizing four steps, based on his experience and observations of training surgical residents [20]. Peyton’s four-step method includes:

3. Demonstration—The entire skill is performed in real time, with limited commentary or teaching.
4. Deconstruction—The teacher repeats the skill while explaining each component of the overall skill in detail.
5. Comprehension—The learner explains each component to the teacher, guiding them to complete the entire skill.
6. Performance—The learner completes the skill while receiving feedback from the teacher [20].

Peyton proposed this method to improve active engagement with the trainee, provide opportunities for immediate feedback, and enable remediation of surgical skills. This approach aligns with our evolving understanding of educational theory for psychomotor skill development. Specifically, Peyton’s four-step method provides contemporaneous feedback, mental practice, and deliberate practice, all shown to improve performance over time [12, 15, 17, 21, 22]. Moreover, recent evidence suggests that Peyton’s four-step approach can lead to better learning outcomes for specific skills in medical education [19].

Peyton’s four-step method has been shown to improve immediate skill acquisition for medical skills; however, its effect on long term skill retention is less clear [19]. In addition, the effect of Peyton’s four-step method on longitudinal skill acquisition in the context of ongoing, required, deliberate skill practice has not been previously evaluated. Furthermore, it has been posited that Peyton’s four-step method is most beneficial for acquiring complex skills, and less so for relatively simple tasks [23]. For POCUS-related skills, use of Peyton’s four-step method in conjunction with a flipped classroom education model has been shown to improve immediate skill acquisition for thyroid and lymph node ultrasound [24]. Additional studies have evaluated the effect of Peyton’s four-step method when teaching echocardiography skills and musculoskeletal ultrasound [25, 26]. Both studies failed to show statistically significant benefit, but notably, both employed peer-to-peer teaching instead of teaching provided by those with expertise. The effect of Peyton’s four-step method on other POCUS applications, when taught by experienced healthcare providers is not well known.

The purpose of this study was to evaluate the effect of Peyton’s four-step method on POCUS image acquisition psychomotor skill development in the setting of ongoing deliberate skill practice. Secondarily, we assessed if the effect of Peyton’s four-step method varied based on POCUS application or the phase of skill acquisition.

## Methods

The study was deemed exempt by the Mayo Clinic institutional review board (24-06851). A single-blinded, repeated measures experimental study was completed. Eligibility criteria included first year PA students from one large academic medical center. Exclusion criteria included students with prior ultrasound experience. Students were notified of classroom research and offered the opportunity to opt out of the study via email to a research coordinator. Faculty were forever blinded to which students opted out of the study; however, upon completion of the study, the primary investigator was notified of how many students opted out for reporting purposes. Students who opted out of the research project would be placed in the control group to receive the standard education provided by the program. Utilizing simple randomization prepared by an independent statistician not involved in participant recruitment or data collection, all participants were randomized into a control group (using Halsted’s two-step method) and intervention group (using Peyton’s four-step method) to assess the influence of Peyton’s four-step approach on POCUS psychomotor skill development. This PA program has a well-established longitudinally integrated POCUS curriculum [1]. Students are assessed through various methods, including knowledge and interpretation-focused quizzes and tests, and hands-on skills examinations. Importantly, the curriculum also includes a POCUS portfolio that requires ongoing deliberate practice of POCUS skills throughout the study period.

To maintain a four-to-one student-to-teacher ratio, six total practicing clinicians (physicians or PAs) with expertise in POCUS were recruited to be educators for the study. All educators were required to be credentialed to utilize POCUS in their local clinical practice and had experience with POCUS education to ensure a baseline level of competency. All educators were allowed to express a preference for which group (control vs. intervention) they wished to teach to maximize educator comfort level with delivery method. Any educator without a preference was randomly assigned to teach using either Peyton’s four-step method or Halsted’s two-step method. A step-by-step “facilitator guide” with directions on how to teach each view for each organ-system was developed and reviewed by all educators for both study groups to ensure standardization of education. Standardization meetings were had for the educators utilizing Peyton’s four-step method to ensure consistent application of the method. Education on aorta, bladder, cardiac, lung, and renal POCUS was provided to students over a two-month period, horizontally aligned with the rest of the PA program curriculum. Each hands-on skill received dedicated education comprised of two hours; first the skill was taught (either utilizing the two-step or four-step method), then followed by learner practice utilizing other colleagues as models. A script of the specific steps to teach each view for the organ-systems was utilized by educators in both the control and intervention groups to support standardization. All students received the same education on cognitive skills; however, psychomotor skills were taught based on their randomization.

**Fig. 1:**
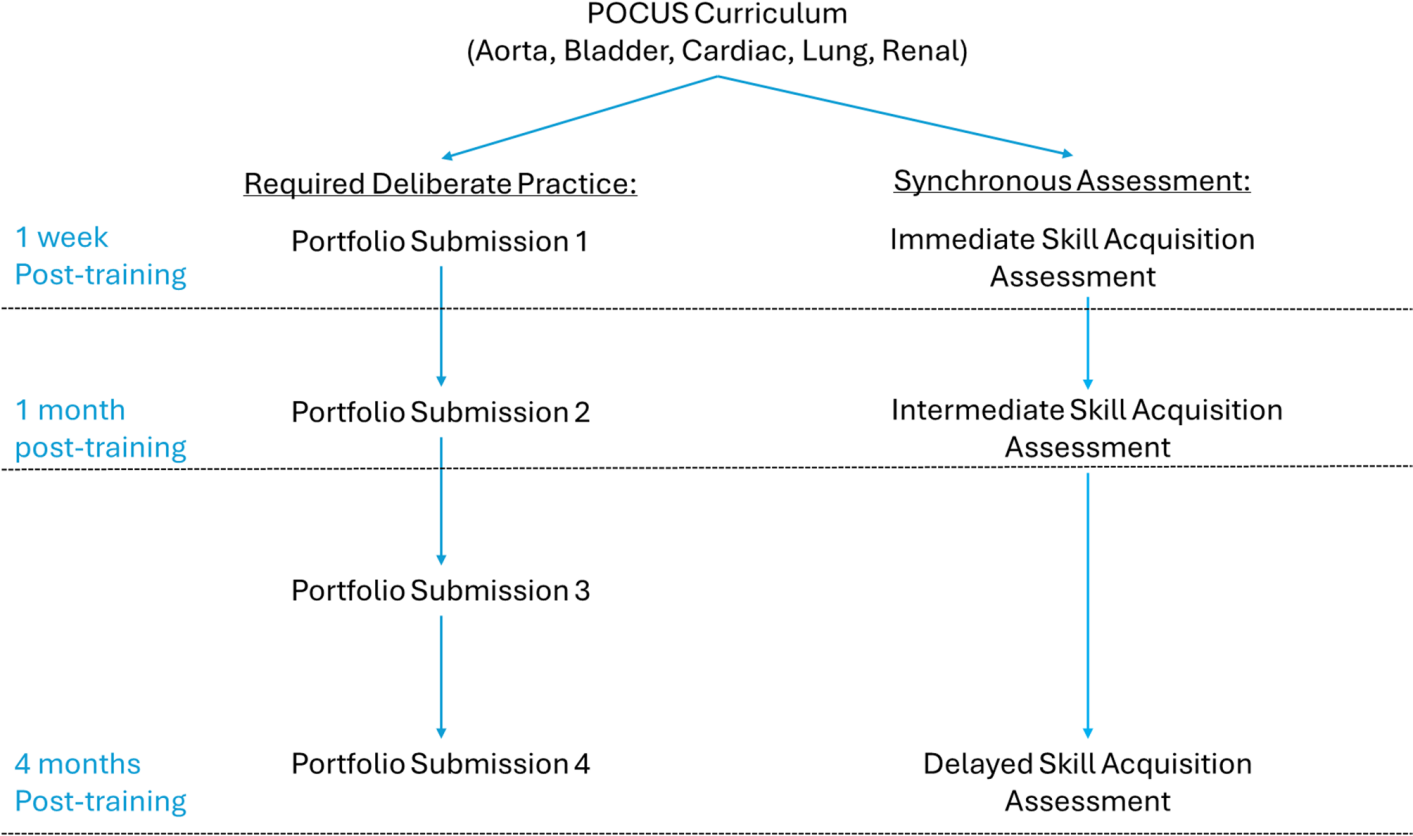
Flow chart of the study design

Following completion of the curriculum, students were assessed via hands-on skills examinations at three different intervals: in the immediate skill acquisition phase (one week after completion of the POCUS curriculum), the intermediate skill acquisition phase (one month after completion of the POCUS curriculum), and the delayed skill acquisition phase (four months after completion of the POCUS curriculum) (Fig. 1). The timing of these assessments was determined to align with the overall PA program curriculum, with the final assessment corresponding to the completion of the didactic phase of the PA program curriculum. Throughout the study, based on the existing curricular design, students were required to have continued deliberate practice of POCUS skills, submitting a minimum of eleven exams (two aorta, bladder, lung, and kidney; three cardiac) prior to the first assessment, an additional fourteen exams (two bladder; three aorta, cardiac, lung, and kidney) prior to the second assessment, and an additional twenty-five exams (four bladder; five aorta, lung, renal; six cardiac) prior to the third assessment. Three additional practicing clinicians with expertise in evaluation of PA student learners and POCUS were recruited to assess learner performance during the synchronous, in-person hands-on skills examination and were blinded to the randomization of the students. Students were assessed using a previously validated instrument titled, the “Observed Structured POCUS Readiness Exam” (OSPRE) [27]. Learners were evaluated on the following criteria: ultrasound device utilization, purposeful examination, image depth, image axis, image gain, overall image quality, structure identification, and interpretation. All criteria are evaluated on a five-point Likert scale from very poor (1) to exemplary (5). The portfolio of POCUS examinations completed as part of the required curriculum to ensure ongoing, deliberate practice, were reviewed contemporaneously. While all examinations were deemed satisfactory, analysis of student performance on these formative examinations was not included in this study. As students select which asynchronous examinations to submit for the portfolio, the synchronous hands-on skills assessment on standardized patients was thought to better represent the students’ skillset.

### Statistical methods

At each assessment, organ specific OSPRE scores were obtained, and a total score was calculated as the sum of the organ specific scores. Scores were summarized for each assessment using median (Q1, Q3) and compared between treatment groups using the Wilcoxon rank sum test. In addition, using data from all assessments an ordinal logistic regression analysis was performed using a generalized linear mixed model with a multinomial distribution and cumulative logit link function. For this model, the independent variable was treatment (Peyton’s four-step method vs. usual instruction), time-period was included as a covariate, and student was included as a random effect. Results from the ordinal logistic regression analyses were summarized by presenting the odds ratio (OR) and 95% lower confidence bound (LCB) for the treatment effect, where odds ratios greater than 1.0 indicate an increased likelihood of higher scores for students who were taught using Peyton’s method versus usual instruction. In all cases, one-tailed p-values were presented with *p* < 0.05 considered statistically significant. As prior literature has demonstrated improved performance for medical skills with Peyton’s four-step method, the research hypothesis was that Peyton’s four-step method improved POCUS performance. As such, a one-tailed Wilcoxon rank sum test and 95% LCB were utilized for statistical analysis.

## Results

No faculty expressed a preference for which group (control versus intervention) they taught, and thus, they were all randomly assigned to a teaching method. The PA program matriculated 25 students into the cohort that participated in the study. Unrelated to the study, one student decelerated from the cohort of learners, and one additional student had prior ultrasound experience. Both students were excluded from the study. Twenty-three of the twenty-three eligible students participated in the study, without any students opting out of the research study. Based on randomization, eleven students were in the control group and twelve were in the intervention group. All 23 students completed the teaching sessions, image portfolio, and assessments associated with the study without any participant crossover or missing data. All twenty-three students completed one examination of each organ system (aorta, bladder, cardiac, lung, and renal) at three individual times, totaling fifteen examinations per student. Over the course of the research project, 345 individual examinations were completed during hands on skills assessments: sixty-nine per organ system.

OSPRE scores are summarized in Table 1. For the purposes of the PA program curriculum, scores were converted to percentages for grades, with all students achieving a passing grade, suggesting overall satisfactory performance. The median (Q1, Q3) total score for students taught using Peyton’s method versus usual instruction was 190 (185, 192) vs. 178 (177, 186) at the immediate assessment, 190 (186, 195) vs. 187 (180, 197) at the intermediate assessment and 198 (185, 199) vs. 193 (188, 195) at the delayed assessment; with significant differences observed at the immediate (*p* = 0.031) and delayed (*p* = 0.011) assessments. From a repeated measures analysis which included data from all assessments, students who were taught using Peyton’s method were found to have an increased likelihood of higher total scores compared to those taught using usual instruction (OR = 4.2, *p* = 0.003). From the repeated measures analyses of the organ-specific OSPRE scores, students taught using Peyton’s method were found to have an increased likelihood of higher scores for cardiac (OR = 2.3, *p* = 0.032), lung (OR = 2.5, *p* = 0.034), and kidney (OR = 3.0, *p* = 0.015). Non-statistically significant improvements were noted for aorta (OR = 2.7, *p* = 0.58) and bladder (OR = 1.7, *p* = 0.121).

**Table 1:** POCUS assessment results*

	Peyton’s method	Usual instruction	
Component	(*N* = 12)	(*N* = 11)	p-value^†^
*Cardiac score*			
Immediate	38 (35, 39)	36 (35, 40)	0.351
Intermediate	37 (36, 39)	35 (34, 38)	0.178
Delayed	40 (39, 40)	38 (34, 40)	0.034
Overall treatment effect^‡^: OR = 2.3, LCB = 1.1, *p* = 0.032			
*Lung score*			
Immediate	38 (33, 39)	35 (33, 39)	0.256
Intermediate	40 (37, 40)	39 (32, 40)	0.199
Delayed	40 (40, 40)	40 (38, 40)	0.028
Overall treatment effect^‡^: OR = 2.5, LCB = 1.1, *p* = 0.034			
*Kidney score*			
Immediate	39 (38, 40)	36 (33, 39)	0.018
Intermediate	39 (39, 40)	39 (38, 40)	0.372
Delayed	40 (38, 40)	39 (37, 40)	0.087
Overall treatment effect^‡^: OR = 3.0, LCB = 1.3, *p* = 0.015			
*Bladder score*			
Immediate	40 (38, 40)	38 (37, 40)	0.169
Intermediate	39 (38, 40)	39 (39, 40)	0.600
Delayed	40 (39, 40)	39 (38, 40)	0.084
Overall treatment effect^‡^: OR = 1.7, LCB = 0.8, *p* = 0.121			
*Aorta score*			
Immediate	38 (37, 40)	38 (35, 39)	0.225
Intermediate	39 (37, 40)	39 (37, 40)	0.395
Delayed	40 (39, 40)	38 (37, 39)	0.009
Overall treatment effect^‡^: OR = 2.7, LCB = 1.0, *p* = 0.058			
*Total score*			
Immediate	190 (185, 192)	178 (177, 186)	0.031
Intermediate	190 (186, 195)	187 (180, 197)	0.298
Delayed	198 (195, 199)	193 (188, 195)	0.011
Overall treatment effect^‡^: OR = 4.2, LCB = 2.0, *p* = 0.003			

*Assessment scores are summarized using median (25th, 75th )

^†^Wilcoxon rank sum test, one-tailed exact p-value

^‡^The overall treatment effect was obtained from an ordinal logistic regression analysis performed using a generalized linear mixed model with a multinomial distribution and cumulative logit link function. For this model, the independent variable was treatment (Peyton’s method vs. usual instruction), time-period was included as a covariate, and student was included as a random effect. Results are summarized by presenting the odds ratio (OR) and 95% lower confidence bound (LCB) for the treatment effect along with the associated one-tailed p-value

## Discussion

Peyton’s four-step method has previously been shown to improve overall psychomotor skill development for specific procedures in medical education [19]. This study further supports this finding and adds to the evidence that this method is more effective for teaching medical skills than the two-step method. Importantly, however, this is the first study to show superiority of this method specifically for teaching psychomotor skills for cardiac, lung, and renal POCUS. In addition, this study expands the literature demonstrating the benefit of this method when used by experts to teach POCUS. Two prior studies have evaluated the effect of Peyton’s four-step method to teach echocardiography skills, a skill adjacent to cardiac POCUS, and musculoskeletal ultrasound skills [25, 26]. However, both studies used a peer-to-peer teaching model and failed to show a statistically significant difference in the teaching modalities. As such, authors have hypothesized that Peyton’s four-step method is most beneficial when utilized by experts [25]. The findings of improved POCUS skill development when educators use Peyton’s four-step method have significant impacts on curricular design and training modalities. Educators of novice POCUS learners should teach POCUS psychomotor skills using Peyton’s four-step method to improve learner performance.

Prior research has not clearly defined the impact of Peyton’s four-step method after the initial skill acquisition phase. In their systematic review and meta-analysis, Giacomino, Caliesch, and Sattelmayer (2020), evaluated the effect of Peyton’s four-step method on skill retention with repeated measures, finding an effect size of a 0.7 standard mean difference (95% CI [– 0.09–1.49]) [19]. Importantly, only five studies included in the systematic review and meta-analysis included repeated measures [28–31]. While a trend towards improvement was noted, it was not found to be statistically significant. Additionally, since the publication of the systematic review and meta-analysis, Peyton’s four-step method had no significant benefit on repeat test after six months for teaching forearm casting when compared to the two-step method [32]. Ours is the first study to assess the effect of Peyton’s four-step method on longitudinal skill acquisition in the setting on ongoing, deliberate practice. In our study, this method had statistically significant benefits in the immediate and delayed skill acquisition phase, but not the intermediate phase. The reason behind these findings remains unclear; however, the difference may relate to the theoretical foundations of motor learning. Fitts and Posner (1967) posited that learners progress through three stages while developing motor skills; (1) The cognitive phase (skills require significant cognitive effort; performance is slow, resulting in many errors, and is based on mimicking movements and trial and error), (2) The associative phase (skills require less cognitive effort, performance improves quickly, errors reduce, and learners can adapt movements more naturally), and (3) The autonomous phase (skills are increasingly autonomous and of high quality, but further improvement ceases or decreases its rate) [16]. We hypothesize that improved performance in the immediate and delayed skill acquisition phases, but not in the intermediate phase represents differing rates of progression through Fitts and Posner’s stages of motor learning. This is important for multiple reasons. The findings of this study suggest that Peyton’s four-step method enhances performance in the cognitive stage, immediately after learners are taught the skill. Additionally, longitudinal skill acquisition for POCUS can be expedited using Peyton’s four-step method. This is demonstrated by the improved performance overall, and specifically in the delayed skill acquisition phase, despite similar amounts of deliberate practice. Theoretically, learners taught with Peyton’s four-step method might require less deliberate practice to progress toward more autonomous performance of a skill. Importantly, learners taught with Halsted’s two-step method can likely achieve similar levels of performance, just at slower rates or after relatively more deliberate practice. This is the first time this benefit has been reported in the literature.

Peyton’s four-step method was originally described as a way of acquiring complex clinical skills pertaining to operative procedures (Peyton, 1998) and prior researchers have hypothesized that it is most beneficial for development of complex skills [20, 23]. Our study supports this hypothesis, as Peyton’s four-step method was not beneficial for bladder POCUS, a relatively easy skill based on prior studies, but was for harder skills, such as cardiac, lung, and renal POCUS [13, 14]. Prior research has shown that the learning curve of aorta POCUS is similar to lung and renal POCUS.^14^ It is notable that in our study, a statistically significant benefit for Peyton’s four-step method on aorta POCUS was not noticed. However, there was a trend toward improvement (OR = 2.7, LCB = 1.0, *p* = 0.058). It is possible that a larger sample would have resulted in a statistically significant finding. This application-specific difference within a singular skill has never been demonstrated before. Educators should prioritize using Peyton’s four-step method for complex medical skills; however, the two-step method is likely sufficient for simple tasks.

This research project has several limitations. The most significant limitation of the study is the small sample size (*n* = 23). While this research study demonstrates several novel findings, generalizability is inherently limited given the small sample size. Thus, larger studies to confirm the results of this study are needed. The sampling methods, and study sample (PA students only) limit the generalizability of the findings. However, novice PA students have similar learning curves to other novice medical learners, therefore it would be reasonable to generalize our findings to other novice learners [13, 14]. Additionally, between assessments, learners may have practiced POCUS skills with individuals taught via the other method, and peer-to-peer teaching during these informal practice sessions is likely. It is unclear how this might affect the results of the study. However, given the results, it is unlikely this would have improved the performance of the intervention group and would have more likely diminished the difference between the groups. Given the nature of the study, educators were aware of the study and unblinded to the method of education they were providing. This has the potential to bias the results of the study, potentially limiting the results. This was mitigated by allowing educators to choose their preferred method, providing standardized instructions, and by having multiple educators for each teaching method.

Given these limitations, further studies with larger samples or multiple learner types should be completed to replicate these findings, increasing generalizability. The final skill assessment in this study occurred after a minimum of eight bladder examinations, ten aorta, lung, and renal examinations, and twelve cardiac examinations. Current evidence suggests that, except for bladder POCUS, psychomotor skill levels generally plateau after fifteen or more examinations, and guidelines recommend a minimum of twenty-five exams be completed per organ system [10, 14]. Future research should assess the long-term effect of the use of Peyton’s four-step method after additional deliberate practice. Research comparing learning curves between students taught with Peyton’s four-step method and the standard, two-step method after completion of the guideline-recommended 25 POCUS examinations, should be considered. Alternatively, performance on a hands-on skills examination, as utilized in this study, could be compared after a prolonged period of deliberate practice, such as at the time of graduation from the PA program.

## Conclusion

Peyton’s four-step method improves overall psychomotor skill acquisition for POCUS. This is the first study to demonstrate that Peyton’s four-step method improves longitudinal performance in the setting of ongoing, deliberate skill practice. This method demonstrated the most significant enhancement of psychomotor skills during the delayed skill acquisition phase, with statistically significant improvements also observed in the immediate skill acquisition phase. The benefit to Peyton’s four-step method was noted in more complex POCUS applications, such as cardiac, lung, and renal POCUS; supporting its use to teach complicated procedures.

## Data Availability

The datasets used and/or analyzed during the current study are available from the corresponding author on reasonable request.

## Abbreviations

OSPRE: Observed Structured POCUS Readiness Exam
PA: Physician Assistant
POCUS: Point-of-Care Ultrasound
